# Virologic success under antiretroviral therapy among people living with HIV/AIDS in the state of Paraná, Brazil

**DOI:** 10.31744/einstein_journal/2021AO5800

**Published:** 2021-11-10

**Authors:** Frederico Alves Dias, Doroteia Aparecida Höfelmann, Yanna Dantas Rattmann

**Affiliations:** 1 Universidade Federal do Paraná Curitiba PR Brazil Universidade Federal do Paraná, Curitiba, PR, Brazil.

**Keywords:** HIV, AIDS, Anti-retroviral agents, Health profile, Sustained virologic response

## Abstract

**Objective:**

To characterize the sociodemographic profile of the population undergoing antiretroviral treatment in the state of Paraná, Brazil, to investigate the proportion of people undergoing treatment among all those diagnosed, and to analyze the proportion of patients with suppressed viral load in different regions of the state.

**Methods:**

Observational descriptive and analytical study carried out with information referring to the period from January 2018 to January 2019. Data were obtained from the *Sistema Informatizado de Monitoramento Clínico das Pessoas Vivendo com HIV/AIDS* [Computerized System for Clinical Monitoring of People Living with HIV/AIDS] and *Sistema de Controle Logístico de Medicamentos*[Drug Supply Control System]. The proportion of people on antiretroviral treatment in the state and the proportion of patients with viral load ≤1,000 copies/mL and ≤50 copies/mL were calculated. The results were compared with the corresponding parameters of the World Health Organization goal 90-90-90.

**Results:**

The state of Paraná managed to reach the second and third parameters of the 90-90-90 goal of the World Health Organization. Among those diagnosed, 93.12% were on antiretroviral treatment, and 90.0% of them had a viral load below 50 copies of viral RNA/mL of blood, indicating virologic success.

**Conclusion:**

The health policy aimed at the population living with HIV/AIDS, and the health services available in Paraná have been successful in parameters relevant to the control of the epidemic. However, it is necessary to ensure the diagnosis of people infected with HIV in the population.

## INTRODUCTION

Viral load suppression is a key marker for effectiveness of antiretroviral therapy, risk of progression of clinical disease and the chance of transmitting the human immunodeficiency virus (HIV).^(1,2)^ Based on this marker, it is possible to guide health planning and efforts to control infection.^(1)^

With this purpose, health systems in different countries follow up the diagnosis and treatment of people living with HIV/AIDS, through actions resulting in a cascade of care, including HIV screening, linkage to care, onset of therapy with antiretrovirals, monitoring patient compliance to drug therapy and, at last, viral suppression.^(1)^

In 2014, the Joint United Nations Programme on HIV/AIDS (UNAIDS) of the World Health Organization (WHO) proposed a target known as 90-90-90, aiming to diagnose 90% of people living with HIV/AIDS, treat 90% of them with antiretroviral drugs, and achieve viral load suppression in 90% of people on treatment. Mathematical models suggest that if these goals are reached by 2020, this could eradicate the global AIDS pandemic by 2030.^(3)^ Brazil was one of the countries in Latin American and the Caribbean to formally commit to the 90-90-90 target.^(4)^

A few computer systems developed by the Ministry of Health are contributing to monitoring of this target. They include the *Sistema de Controle Logístico de Medicamentos* (SICLOM) [Drug Supply Control System], which, among other functionalities, monitors dispensing of antiretroviral therapies. There is also the *Sistema de Monitoramento Clínico das Pessoas Vivendo com* HIV/AIDS (SIMC) [System for Clinical Monitoring of People Living with HIV/AIDS], which monitors the number of people diagnosed with HIV/AIDS, and those who already have a diagnosis however have not yet initiated antiretroviral therapy (treatment gap), in addition to providing access to laboratory test results.^(5)^

## OBJECTIVE

To characterize the profile of the population in antiretroviral therapy in the state of Paraná, investigate the proportion of people on therapy among all patients diagnosed (treatment gap), and assess the percentage of patients with suppressed viral load.

## METHODS

This is a descriptive, observational investigation of sociodemographic and treatment gap information, and an analytical, observational investigation of viral load information of patients on therapy.

The sociodemographic profile of people on antiretroviral therapy in the state of Paraná was obtained from the SICLOM system. The variables of interest were sex, age group, skin color, and years of education.

Antiretroviral treatment gap information was collected in January 2019, from the SIMC system. The number of people diagnosed with HIV in the state and the number of people on therapy allowed for calculation of the treatment gap in this population.

Viral load data of people living with HIV/AIDS in the state of Paraná were also obtained from the SIMC database, between January 2018 to January 2019, and monitored bimonthly. This study considered only patients who were on antiretroviral therapy for at least 6 years before data collection, and with at least one antiretroviral drug dispensing in the 100 days preceding data collection, as defined in the Clinical Protocol and Therapeutic Guidelines for HIV infection management in adult patients of the Ministry of Health.^(5)^

Treatment gap information and viral load results of the state of Paraná were compared with the provisions of the UNAIDS/WHO 90-90-90 target.^(3)^

To assess the viral load suppression rate in the state of Paraná, the first step was to quantify the number of patients on antiretroviral therapy in the state, and in each of its 22 Regional Health Divisions (RS - *Regionais de Saúde*). These data were obtained from the SICLOM system. Next, the proportion of patients with viral load ≤1,000 copies/mL and ≤50 copies/mL was determined for the period between January 2018 and January 2019, at every two months. The study analyses considered the cutoff of 1,000 copies/mL based on the global UNAIDS/WHO target,^(3)^ and 50 copies/mL based on the Clinical Protocol and Therapeutic Guidelines.^(5)^

Administratively wise, the state of Paraná is divided in 22 RS, and their head offices are located in regional hub cities. These regional divisions are identified as: 1^st^ RS – Paranáguá, 2^nd^ RS – Metropolitana de Curitiba, 3^rd^ RS – Ponta Grossa, 4^th^ RS – Irati, 5^th^ RS – Guarapuava, 6^th^ RS – União da Vitória, 7^th^ RS – Pato Branco, 8^th^ RS – Francisco Beltrão, 9^th^ RS – Foz do Iguaçu, 10^th^ RS – Cascavel, 11^th^ RS – Campo Mourão, 12^th^ RS – Umuarama, 13^th^ RS – Cianorte, 14^th^ RS – Paranavaí, 15^th^ RS – Maringá, 16^th^ RS – Apucarana, 17^th^ RS – Londrina, 18^th^ RS – Cornélio Procópio, 19^th^ RS – Jacarezinho, 20^th^ RS – Toledo, 21^st^ RS – Telêmaco Borba, and 22^nd^ RS – Ivaiporã.^(6)^

The 2^nd^ RS comprises 29 cities, including Curitiba.^(6)^ However, data on the services managed by the City Administration were obtained and reviewed independently. This is because Curitiba is a self-managed municipality reporting directly to the State Coordination for Sexually Transmitted Infections/AIDS and, oftentimes, directly to the Ministry of Health, and its data are provided separately on the system.^(6)^ Therefore, the results of the 2^nd^ RS include information on the other 28 cities.

In the statistical analysis, the hypothesis considered was first-order autocorrelation between the proportions observed, due to the fact this was virtually the same population being monitored over time. This analysis used the Durbin-Watson test and proportions were corrected by the Prais-Winsten estimation.

With the view to estimate the increase or decrease in the viral load suppression rate over the course of the study period, the estimated variation percentage was calculated based on the linear regression adjusted to the natural log of proportions. Proportion variations were considered significant when p≤0.05. The statistical package used was the Stata version 14.

The study was approved by the Research Ethics Committee of the *Universidade Federal do Paraná* (UFPR), under opinion 2.620.673/2018, CAAE: 82936318.3.0000.0102, and the Research Ethics Committee of the *Hospital do Trabalhador/Secretaria do Estado da Saúde do Paraná*, under opinion 2.674.606/2018; CAAE: 82936318.3.3001.5225. Since this study was based on secondary data, it was exempt from the need to sign an Informed Consent Form (ICF).

## RESULTS

People living with HIV/AIDS in the state of Paraná during the study period were predominantly men (60.0%), aged 30 to 49 years (49.5%), white (61.6%) and with 4 to 11 (51.2%) years of study (Table 1). The ratio of men to women was 1.5.

**Table 1:** Sociodemographic characteristics of the population on antiretroviral therapy in the state of Paraná

Variables		n (%)
Sex		
	Male	19,241 (60.0)
	Female	12,822 (40.0)
	Not reported	33 (0.1)
Age group, years		
	<5	1,069 (3.3)
	5-19	650 (2.0)
	20-29	5,083 (15.8)
	30-39	7,651 (23.8)
	40-49	8,250 (25.7)
	50-59	6,221 (19.4)
	>60	3,172 (9.9)
Skin color		
	White	19,783 (61.6)
	Brown	5,112 (15.9)
	Black	1,403 (4.7)
	Yellow	140 (0.4)
	Indigenous	31 (0.1)
	Not reported	5,627 (17.5)
Education, years		
	None	1,569 (4.9)
	1-3	2,211 (6.9)
	4-7	8,147 (25.4)
	8-11	8,285 (25.8)
	or more	3,985 (12.4)
	Not reported	7,899 (24.6)
Total		32,096 (100.0)

In January 2019, the end of the study period, 34,472 people had been diagnosed with HIV in the state of Paraná, 93.12% of which were on antiretroviral therapy. The other 2,376 were in a treatment gap and had not yet initiated antiretroviral therapy.

In January 2018, the start of the observation study, 91.4% of people on antiretroviral therapy already had viral load ≤1,000 copies/mL in the state of Paraná (93.3±2.0%). However, there are important differences in viral load values among the different Regional Health Divisions in the state (Table 2).

**Table 2:** Percentage of patients with viral load ≤1,000 copies/mL, regression coefficients and variations in the state of Paraná and Regional Health Divisions, between January 2018 and January 2019

Regional Health Division (RS)	2018 (%)						2019 (%)	Coefficient	95%CI	p value	Variation (%)	95%CI	Interpretation
	January	March	May	July	September	November	January						
1^st^ Paranaguá	87.7	87.8	88.3	92.0	92.3	93.5	94.4	1.38	0.9-1.8	0.001	3.55	2.86-4.24	Increase
2^nd^ Metropolitana de Curitiba	92.2	91.8	92.5	94.9	95.1	96.1	96.9	1.00	0.7-1.3	<0.001	2.48	2.06-2.90	Increase
3^rd^ Ponta Grossa	89.0	89.0	88.8	91.2	92.0	93.2	95.6	1.46	0.9-2.0	0.002	3.66	2.77-4.55	Increase
4^th^ Irati	94.5	93.3	91.4	94.9	94.5	95.0	95.9	0.61	-0.0-1.2	0.055	1.51	0.54-2.49	Stability
5^th^ Guarapuava	87.5	88.2	88.5	92.1	93.5	94.2	94.7	1.46	0.9-2.0	0.002	3.74	2.78-4.72	Increase
6^th^ União da Vitória	87.0	86.5	87.0	92.6	92.1	94.7	95.6	1.89	1.3-2.5	0.001	4.91	3.94-5.89	Increase
7^th^ Pato Branco	86.3	86.1	85.4	90.4	91.3	93.8	94.8	1.95	1.2-2.7	0.002	5.11	3.94-6.30	Increase
8^th^ Francisco Beltrão	91.7	91.4	92.2	93.5	93.9	95.7	97.2	1.23	0.8-1.6	0.001	2.98	2.40-3.56	Increase
9^th^ Foz do Iguaçu	92.1	91.6	92.8	94.4	94.9	96.3	97.1	1.05	0.9-1.2	<0.001	2.59	2.41-2.77	Increase
10^th^ Cascavel	91.9	91.4	92.0	93.8	93.6	93.5	96.9	0.72	0.4-1.0	0.004	1.80	1.29-2.31	Increase
11^th^ Campo Mourão	88.2	87.9	88.1	91.8	92.5	93.9	94.0	1.38	0.8-1.9	0.002	3.55	2.71-4.40	Increase
12^th^ Umuarama	90.4	89.9	91.0	94.7	94.7	95.2	95.7	1.18	0.5-1.8	0.008	2.97	1.91-4.04	Increase
13^th^ Cianorte	76.8	77.0	80.2	90.2	87.6	88.2	91.9	2.75	0.7-4.7	0.019	7.81	4.1-11.6	Increase
14^th^ Paranavaí	92.5	92.6	92.9	95.0	93.6	94.6	97.5	0.67	0.2-1.1	0.011	1.65	1.0-2.3	Increase
15^th^ Maringá	91.1	91.2	92.6	94.0	93.3	94.0	96.5	0.79	0.4-1.2	0.004	1.98	1.4-2.6	Increase
16^th^ Apucarana	85.5	84.7	84.9	90.1	89.9	91.6	95.2	1.95	1.3-2.6	0.001	5.17	4.1-6.2	Increase
17^th^ Londrina	89.8	89.4	89.9	92.0	93.0	93.5	95.8	1.23	0.9-1.5	<0.001	3.11	2.6-3.6	Increase
18^th^ Cornélio Procópio	84.8	84.9	83.7	87.1	85.9	89.9	93.2	1.86	0.4-3.3	0.023	4.83	2.6-7.1	Increase
19^th^ Jacarezinho	83.2	82.7	82.4	89.5	89.0	90.2	92.9	2.02	1.2-2.9	0.003	5.51	4.0-7.0	Increase
20^th^ Toledo	86.5	85.6	86.5	88.8	87.6	91.3	94.0	1.61	0.7-2.5	0.007	4.16	2.8-5.5	Increase
21^st^ Telêmaco Borba	85.4	85.5	86.6	89.0	88.6	88.7	89.5	0.72	0.1-1.3	0.028	1.91	0.9-2.9	Increase
22^nd^ Ivaiporã	84.8	83.6	84.2	88.5	88.9	91.7	93.6	2.05	1.5-2.6	<0.001	5.46	4.6-6.3	Increase
City Administration of Curitiba	94.9	94.5	94.9	95.8	96.1	96.7	97.7	0.65	0.5-0.8	<0.001	1.56	1.3-1.8	Increase
Paraná	91.4	91.1	91.6	93.8	94.0	94.9	96.4	1.01	0.8-1.2	<0.001	2.52	2.1-2.9	Increase

Durbin-Watson test and ratios corrected by Prais-Winsten statistics. p values ≤ 0.05 were considered significant.

95%CI: 95% confidence interval.

**Figure 1:**
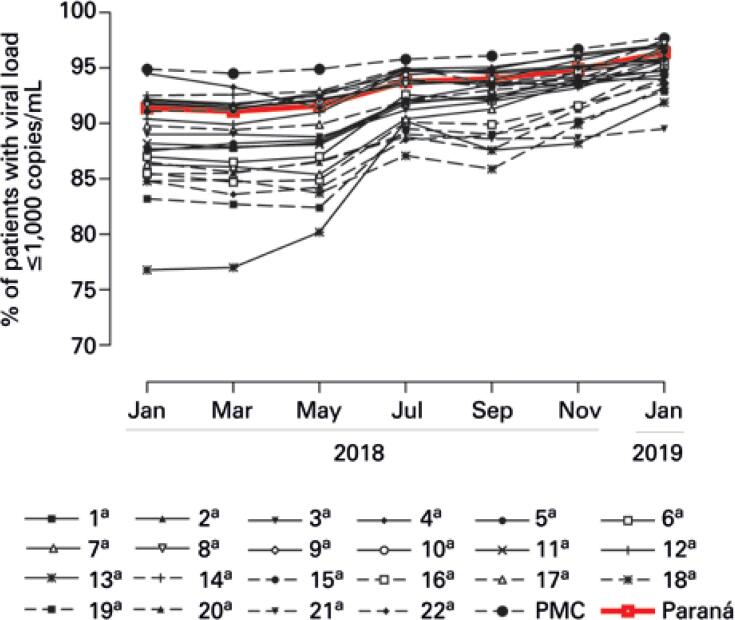
Evolution of the rate of patients with viral load ≤1,000 copies/mL in the state of Paraná and its Regional Health Divisions, between January 2018 and January 2019

The highest mean of patients with viral load under 1,000 copies/mL was obtained by the Regional Health Division of the City Administration of Curitiba (95.8±1.1%), whereas the lowest mean was in the 13^th^ RS (84.5±6.4%). Table 2 shows the bimonthly rate of patients on antiretroviral therapy ≤1,000 copies/mL in all RS of the state of Paraná. Figure 1 illustrates the evolution of Regional Health Divisions and the state for this indicator.

PMC: Prefeitura Municipal de Curitiba.

Regression coefficients showed an increasing trend for the percentage of people with viral load suppression in virtually all RS (p<0.05), except for the 4^th^ RS, which showed a stability trend (p=0.055), and where the rate of patients with viral load ≤1,000 copies/mL exceeded the state mean.

**Figure 2:**
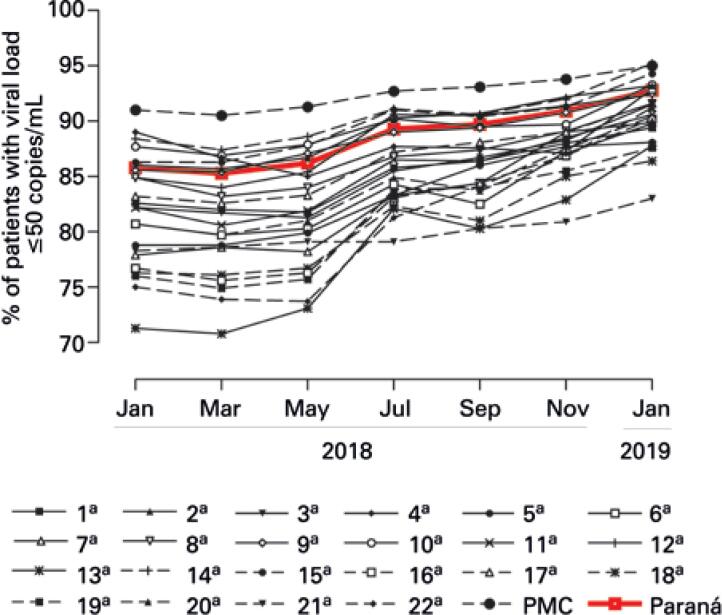
Evolution of the rate of patients with viral load ≤50 copies/mL in the state of Paraná and its Regional Health Divisions, between January 2018 and January 2019

In the viral load ≤50 copies/mL analysis, Paraná achieved 90% of viral load suppression in people on antiretroviral therapy in November 2018, close to the end of the observation period (January 2019). However, considering the entire study period, the mean was below 90% (88.6±2.9%). The highest mean of patients with viral load under 50 copies/mL was obtained by the Regional Health Division of the City Administration of Curitiba (92.5±1.6%), whereas the lowest mean was in the 13^th^ RS (78.3±6.6%) (Table 3). Figure 2 illustrates the evolution of Regional Health Divisions and the state in respect to this indicator.

**Table 3:** Percentage of patients with viral load ≤50 copies/mL, regression coefficients and variations in the state of Paraná and Regional Health Divisions, between January 2018 and January 2019

Regional Health Division (RS)	2018 (%)						2019 (%)	Coefficient	95%CI	p value	Variation (%)	95%CI	Interpretation
	January	March	May	July	September	November	January						
1^st^ Paranaguá	82.6	82.0	81.8	85.8	86.4	88.0	89.4	1.56	1.1-2.1	0.001	4.30	3.4-5.2	Increase
2^nd^ Metropolitana de Curitiba	86.0	85.7	86.7	90.3	90.7	92.2	93.2	1.53	1.1-1.9	<0.001	4.02	3.3-4.7	Increase
3^rd^ Ponta Grossa	82.2	81.7	81.3	85.5	86.7	88.3	91.5	2.08	1.4-2.8	0.001	5.68	4.5-6.9	Increase
4^th^ Irati	89.0	86.7	85.0	87.7	87.6	87.1	89.8	0.67	-0.1-1.5	0.081	1.77	0.5-3.1	Stability
5^th^ Guarapuava	78.8	78.8	79.9	83.3	86.0	87.7	88.1	2.07	1.4-2.7	0.001	5.88	4.7-7.0	Increase
6^th^ União da Vitória	80.7	79.7	80.3	84.3	82.5	87.3	92.9	2.74	0.9-4.6	0.014	7.27	4.4-10.2	Increase
7^th^ Pato Branco	77.9	78.6	78.2	83.3	84.4	89.0	89.9	2.54	1.8-3.3	0.001	7.21	5.9-8.6	Increase
8^th^ Francisco Beltrão	84.9	83.2	84.0	86.8	87.4	89.1	91.6	1.69	1.3-2.1	<0.001	4.52	3.9-5.2	Increase
9^th^ Foz do Iguaçu	85.6	85.4	87.1	89.0	89.4	90.8	92.5	1.29	1.1-1.5	<0.001	3.41	3.1-3.7	Increase
10^th^ Cascavel	87.7	86.9	87.9	90.2	89.5	89.7	93.3	0.88	0.4-1.3	0.005	2.30	1.6-3.0	Increase
11^th^ Campo Mourão	82.2	80.6	81.9	86.5	86.4	88.5	90.6	1.91	1.4-2.4	<0.001	5.28	4.4-6.2	Increase
12^th^ Umuarama	84.9	84.0	85.4	90.6	90.5	91.4	92.4	1.70	0.9-2.5	0.005	4.55	3.1-6.0	Increase
13^th^ Cianorte	71.3	70.8	73.1	82.1	80.3	82.9	87.8	3.08	2.0-4.2	0.001	9.53	7.3-11.8	Increase
14^th^ Paranavaí	88.4	87.4	88.6	91.1	90.6	92.0	95.2	1.29	0.8-1.8	0.002	3.32	2.6-4.1	Increase
15^th^ Maringá	86.3	86.3	87.8	91.0	90.4	91.3	94.3	1.34	0.9-1.8	0.001	3.51	2.8-4.3	Increase
16^th^ Apucarana	76.7	75.6	76.3	83.1	84.4	86.9	90.2	2.98	2.2-3.8	<0.001	8.68	7.1-10.2	Increase
17^th^ Londrina	83.2	82.6	83.3	87.2	88.1	89.0	91.6	1.74	1.3-2.2	<0.001	4.73	3.9-5.5	Increase
18^th^ Cornélio Procópio	76.3	76.1	76.7	82.3	81.0	85.0	86.4	2.04	1.5-2.5	<0.001	5.98	5.0-6.9	Increase
19^th^ Jacarezinho	76.0	74.9	75.7	83.8	84.0	85.5	87.6	2.61	1.5-3.7	0.003	7.72	5.6-9.9	Increase
20^th^ Toledo	80.7	79.7	81.0	84.9	83.7	86.8	91.2	1.95	1.2-2.7	0.002	5.39	4.1-6.7	Increase
21^st^ Telêmaco Borba	78.3	78.6	79.1	79.1	80.3	80.9	83.0	1.86	0.3-3.4	0.030	5.25	2.5-8.0	Increase
22^nd^ Ivaiporã	75.0	73.9	73.7	81.3	84.1	87.5	90.4	3.65	2.5-4.8	0.001	10.80	8.6-13.1	Increase
City Administration of Curitiba	91.0	90.5	91.3	92.7	93.1	93.8	95.0	0.84	0.7-1.0	<0.001	2.12	1.9-2.3	Increase
Paraná	85.8	85.3	86.2	89.3	89.7	90.9	92.8	1.44	1.1-1.8	<0.001	3.79	3.3-4.3	Increase

Durbin-Watson test and ratios corrected by Prais-Winsten statistics. p values ≤0.05 were considered significant.

95%CI: 95% confidence interval.

PMC: Prefeitura Municipal de Curitiba.

The proportion of patients with viral load ≤50 copies/mL increased significantly in the state of Paraná (p<0.001), in Curitiba (p<0.001) and in 21^st^ RS. Except for the 4^th^ RS of the state (p=0.081), of which values (87.6±1.6%) approximated the state mean (88.6±2.9%). There was a 3.79% (3.25%-4.34%) variation in Paraná, pointing to a lower homogeneity of results when compared with the previous rate (≤1,000 copies/mL). A regression coefficient of 1.44 indicates an increasing trend of the rate in the state (p<0.001).

## DISCUSSION

With the purpose of verifying compliance with the UNAIDS/WHO 90-90-90 target,^(3)^ this study investigated the number of people diagnosed with HIV in the state of Paraná, the percentage of people on antiretroviral therapy and, among the latter, the percentage of people with suppressed viral load.

Among 32,096 individuals on antiretroviral therapy in the state of Paraná during the study period, most were male (59.95%), in consonance with other studies.^(7,8)^ The ratio between sexes in the treated population was 15 men for every 10 women (males:females = 1.5). When comparing state and national data of HIV-diagnosed population, this ratio is 21 men for every 10 women (males:females = 2.1) in the state of Paraná, and 26 men for every 10 women (males:females = 2.6) in Brazil.^(9)^ Therefore, the ratio among those diagnosed is not maintained among those on antiretroviral therapy, with a greater disadvantage for the male sex. Considering the recommendation to treat all those diagnosed, this difference may point to difficulties in access or adherence to antiretroviral therapy by men.^(9)^ According to UNAIDS, men are less likely to undergo HIV screening and seek antiretroviral treatment. The causes for these behaviors are not yet fully understood, however they seem to involve empirical masculinity-related concepts.^(10)^

The predominant age group in the antiretroviral therapy population in the state of Paraná was 30-49 years (49.54%), as identified in a previous study.^(11)^ However, in Brazil and Paraná, new cases of HIV infection occur predominantly among people aged 20 to 39 years.^(9,12)^ This age group is, overall, more susceptible to high-risk behaviors and infection by HIV, and this may represent a slight change in the age group of people being treated for HIV.^(12)^

There was also a prevalence of white skin color (61.64%), followed by brown skin color (15.93%). This result was confirmed by other studies and is in consonance with the demographic characteristics of the population of Paraná.^(12-14)^

As for schooling level, the high percentage of ignored cases (24.6%) hindered a thorough evaluation, however more than half of the population on antiretroviral treatment in Paraná has 4 to 11 years of education, similarly to other studies in Paraná and Brazil.^(9,12)^

Brazil has been globally recognized by its strong HIV/AIDS control policy. As a result, the country had already diagnosed 85% of all people living with HIV in its territory in 2017,^(15)^ *i.e*. similarly to the numbers achieved by the state of Paraná, in 2015.^(16)^

Based on information obtained from the *Sistema de Controle de Exames Laboratoriais*(SISCEL) [Laboratory Test Control System] and SICLOM systems, the proportion of people diagnosed with HIV and on antiretroviral therapy was 93.12% of all those diagnosed in the state of Paraná. This means that, during the study period, the state had already achieved the second UNAIDS target of providing therapy to 90% of HIV-diagnosed population.^(3)^ It is worth noting that the Ministry of Health recommends early initiation of antiretroviral therapy, right after a diagnosis of HIV infection, regardless of the clinical and immunological status of infected people, due to the benefits of reducing morbidity and mortality, decreasing transmission and reducing burden of tuberculosis, in addition to the availability of more convenient and well-tolerated therapeutic options.^(5)^ Hence, early initiation of antiretroviral therapy decreases the treatment gap throughout the country.

Several studies support the need to offer antiretroviral treatment to the infected population. A study from South Africa has shown that a 1% increase in antiretroviral therapy coverage led to a 1.1% reduction in HIV incidence.^(17)^ Also, a Canadian study had shown a 1.2% reduction in HIV incidence for every 1% of increase in the number of people with suppressed viral load as a result from therapy.^(18)^

Considering the cutoff of 1,000 viral RNA copies/mL of blood, proposed by UNAIDS/WHO,^(3)^ it was verified the state of Paraná had achieved the target of 90% of people on treatment with suppressed viral load since the start of the study period (January 2018). This level was maintained during one year of the study, and the increasing trend was statistically confirmed.

And considering the most critical cutoff point, proposed by the Clinical Protocol and Therapeutic Guidelines of the Ministry of Health,^(5)^ the percentage of patients with viral load ≤50 viral RNA copies/mL of blood was achieved more recently in the state (November 2018); however, an increasing trend has been identified in Paraná. These numbers suggest positive results of the measures to accelerate HIV control, as well as the success of antiretroviral therapy in the population of Paraná. These successes are likely due to the recommendation to treat all people living with HIV, regardless of immunological status, and the provision of systems to facilitate patient monitoring (such as the SIMC) and incentives from the State Health Department of Paraná for the use of these systems and prescription of more potent regimens since the first line of antiretroviral treatment, including new agents, such as dolutegravir.^(5,19,20)^

In 2015, epidemiological data of the Ministry of Health already showed that Brazil will be successful in achieving the 90% viral load suppression parameter established in the 90-90-90 target. In 2016, the country had 91% of people on antiretroviral therapy with viral load results under 1,000 copies/mL. In 2017, this rate increased to 92%, and this is similar to the results found in the current study for the state of Paraná.^(9)^

However, studies demonstrating the non-transmission of HIV by patients with suppressed viral load adopted a cutoff viral load under 200 copies/mL.^(2)^ In addition, recent studies have shown that a large proportion of patients with viral load under 1,000 copies/mL, however over 80 copies/mL, have mutations that confer resistance to antiretroviral drugs, which can lead to therapy failure and transmission of resistant viruses.^(21)^

Therefore, the Brazilian Ministry of Health considers as virological success patients with viral load under 50 copies/mL, meaning suppressed viral replication with no virus transmission or disease progression.^(22)^

In Paraná, the services provided by the City Administration of Curitiba, the Regional Health Divisions of Paranavaí (14^th^ RS) and Maringá (15^th^ RS) and Metropolitana de Curitiba (2^nd^ RS) achieved the best virologic success results of the state during the study period. However, some RS are still far from the desired rate, such as Cianorte (13^th^ RS), Telêmaco Borba (21^st^ RS) and Cornélio Procópio (18^th^ RS).

These results can be at least partially explained by the differences in health services structuring and socioeconomic factors (such as schooling and income level) of each Regional Health Division. These factors lead to different levels of vulnerability impacting access and adherence to antiretroviral therapy and, subsequently, virologic success rates of the treatment. This relation was identified in several studies.^(21-26)^

Despite differences within the state, regression coefficients revealed an increasing trend for the percentage of people with undetectable viral load in nearly all RS, except for the 4^th^ RS, which showed a stability trend. Other studies had similar^(27,28)^ or lower^(29,30)^ viral load suppression results. This shows that, overall, the health policy and services provided in the state of Paraná have accomplished virological success in people on antiretroviral therapy.

It is worth highlighting that, after reaching 90% viral load suppression in patients on antiretroviral therapy, their viral load must remain suppressed to reduce HIV transmission, which translates into public health benefits.^(20,22)^

It is also worth noting this study considered in its evaluation the second and third stages of the 90-90-90 target, which are, respectively, the percentage of patients on treatment, and patients in viral load suppression. However, the HIV cascade of care contemplates interventions that depend on one another, so that if there is no advance in the diagnosis parameter, the first stage of the goal, the other points of the cascade will not represent reality. For this reason, it is important to monitor all other points in the cascade of continuous HIV care. In this context, it is estimated that 80% of people living with HIV in Brazil know they are infected, but there is no specific information about the state of Paraná.^(15)^

## CONCLUSION

The state of Paraná has reached the second and third parameters of the 90-90-90 target of the World Health Organization’s Joint United Nations Programme on HIV/AIDS (UNAIDS). This shows the health policy targeted at people living with HIV/AIDS and the health services provided in Paraná, despite regional differences, have been successful in parameters relevant to controlling the epidemic. However, these parameters are interdependent and key to reaching and maintaining the first parameter of the target, which is diagnosing 90% of people with HIV in the population. This would require better structuring of the Care Network, which entails investments in health education and strategies to increase demand for HIV screening and counseling services.
