# Supplementary material for: Virologic success under antiretroviral therapy among people living with HIV/AIDS in the state of Paraná, Brazil
Source: Einstein (Sao Paulo). 2021 Nov 10;19:eAO5800. doi: 10.31744/einstein_journal/2021AO5800 (PMC8687700; doi:10.31744/einstein_journal/2021AO5800)
Supplement: Translation

# Translation: Avaliação do sucesso virológico da terapia antirretroviral em pessoas vivendo com HIV/AIDS no estado do Paraná, Brasil

**Authors:** Frederico Alves Dias, Doroteia Aparecida Höfelmann, Yanna Dantas Rattmann

**Affiliations:** 1 Curitiba PR Brasil Universidade Federal do Paraná, Curitiba, PR, Brasil.

**Keywords:** HIV, AIDS, Antirretrovirais, Perfil de saúde, Resposta viral sustentada

## Abstract

**Objetivo:**

Caracterizar o perfil sociodemográfico da população em tratamento antirretroviral no estado do Paraná, investigar a proporção de pessoas em tratamento entre todos os diagnosticados e analisar a proporção de pacientes com carga viral suprimida nas diferentes regiões do estado.

**Métodos:**

Estudo observacional descritivo e analítico realizado com informações referentes ao período de janeiro de 2018 a janeiro de 2019. Os dados foram obtidos do Sistema Informatizado de Monitoramento Clínico das Pessoas Vivendo com HIV/AIDS e do Sistema de Controle Logístico de Medicamentos. Foram calculadas as proporções de pessoas em tratamento antirretroviral no estado e de pacientes com carga viral ≤1.000 cópias/mL e ≤50 cópias/mL. Os resultados foram comparados com os parâmetros correspondentes da meta 90-90-90 da Organização Mundial da Saúde.

**Resultados:**

O estado do Paraná alcançou o segundo e o terceiro parâmetros da meta 90-90-90 da Organização Mundial da Saúde. Entre os diagnosticados, 93,12% encontravam-se em tratamento antirretroviral, e 90,0% destes apresentavam carga viral abaixo 50 cópias do RNA viral/mL de sangue, indicando sucesso virológico.

**Conclusão:**

A política de saúde voltada à população vivendo com HIV/AIDS e os serviços de saúde disponibilizados no Paraná têm obtido êxito em parâmetros relevantes para o controle da epidemia. Entretanto, é necessário assegurar o diagnóstico das pessoas infectadas por HIV na população.

## INTRODUÇÃO

A supressão viral é um marcador crítico da efetividade da terapia antirretroviral, do risco de progressão da doença clínica e da possibilidade de transmissão do vírus da imunodeficiência adquirida (HIV).^(1,2)^ A partir desse marcador, é possível nortear o planejamento em saúde e empreender esforços para o controle da infecção.^(1)^

Com esse propósito, os sistemas de saúde de diversos países monitoram o diagnóstico e acompanham o tratamento das pessoas vivendo com HIV/AIDS, por meio de ações inseridas numa cascata de cuidado, a qual inclui o teste para HIV, a vinculação ao serviço de saúde, o início da terapia com os antirretrovirais e o acompanhamento da adesão aos medicamentos e, por fim, da supressão viral.^(1)^

Em 2014, o Programa Conjunto das Nações Unidas sobre HIV/AIDS (UNAIDS), da Organização Mundial da Saúde (OMS), propôs uma meta conhecida como 90-90-90, que consiste em diagnosticar 90% das pessoas vivendo com HIV/AIDS, tratar 90% delas com medicamentos antirretrovirais e alcançar a supressão viral em 90% das pessoas em tratamento. Os modelos matemáticos sugerem que o alcance desses objetivos até o ano de 2020 erradicaria a epidemia mundial de AIDS até 2030.^(3)^ O Brasil foi um dos primeiros países da América Latina e Caribe a adotar formalmente o compromisso com a meta 90-90-90.^(4)^

Alguns sistemas informatizados foram desenvolvidos pelo Ministério da Saúde e contribuem com o monitoramento dessa meta. Entre eles, está o Sistema de Controle Logístico de Medicamentos (SICLOM), que, entre outras funções, permite acompanhar as dispensações de medicamentos antirretrovirais. Existe ainda o Sistema de Monitoramento Clínico das Pessoas Vivendo com HIV/AIDS (SIMC), o qual permite monitorar o número de pessoas diagnosticadas com HIV/AIDS e de pessoas diagnosticadas que ainda não iniciaram o tratamento antirretroviral (*gap* de tratamento), além do acesso a resultados de exames laboratoriais.^(5)^

## OBJETIVO

Caracterizar o perfil da população em tratamento antirretroviral no estado do Paraná, investigar a proporção de pessoas em tratamento entre todos os pacientes diagnosticados (*gap* de tratamento) e analisar a percentagem de pacientes com carga viral suprimida.

## MÉTODOS

Trata-se de estudo observacional descritivo, no que se refere às informações sociodemográficas e ao *gap* de tratamento, e observacional analítico, no que se refere às cargas virais dos pacientes em terapia.

O perfil sociodemigráfico das pessoas em tratamento antirretroviral do estado do Paraná foi obtido por meio do SICLOM. As variáveis de interesse foram sexo, faixa etária, cor da pele e escolaridade.

As informações sobre o *gap* de tratamento antirretroviral foram coletadas em janeiro de 2019, por meio do SIMC. A quantidade de pessoas diagnosticadas com o HIV no estado e o número de pessoas em tratamento permitiram o cálculo do percentual da população que se encontrava em *gap* de tratamento.

As informações sobre a carga viral das pessoas vivendo com HIV/AIDS no Paraná também foram obtidas a partir da base de dados do SIMC, no período entre janeiro de 2018 a janeiro de 2019, acompanhadas bimestralmente. Foram considerados apenas os resultados dos pacientes em tratamento antirretroviral há, pelo menos, 6 meses antes da data da coleta e que possuíam, no mínimo, uma dispensação de antirretrovirais nos 100 dias anteriores à data da coleta, conforme definido no Protocolo Clínico e Diretrizes Terapêuticas (PCDT) para manejo da infecção pelo HIV em adultos do Ministério da Saúde.^(5)^

Os resultados do *gap* de tratamento e de carga viral do estado do Paraná foram comparados com o preconizado pela meta 90-90-90 da UNAIDS/OMS.^(3)^

Para análise do índice de pacientes com carga viral suprimida no estado do Paraná, primeiramente foi quantificado o número de pacientes em terapia antirretroviral no estado e em cada uma das suas 22 Regionais de Saúde (RS). Esse dado foi obtido por meio do SICLOM. Na sequência, foi determinada a proporção de pacientes com resultado de carga viral ≤1.000 cópias/mL e ≤50 cópias/mL, no período de janeiro 2018 a janeiro de 2019, a cada 2 meses. As análises consideraram os valores de 1.000 cópias/mL, com base na meta mundial da UNAIDS/OMS,^(3)^ e 50 cópias/mL, com base no PCDT.^(5)^

Administrativamente, o estado do Paraná é dividido em 22 RS, cujas sedes administrativas estão localizadas nas cidades-polo da região. Essas regionais são identificada como: 1ª RS – Paranáguá, 2ª RS – Metropolitana de Curitiba, 3ª RS – Ponta Grossa, 4ª RS – Irati, 5ª RS – Guarapuava, 6ª RS – União da Vitória, 7ª RS – Pato Branco, 8ª RS – Francisco Beltrão, 9ª RS – Foz do Iguaçu, 10ª RS – Cascavel, 11ª RS – Campo Mourão, 12ª RS – Umuarama, 13ª RS – Cianorte, 14ª RS – Paranavaí, 15ª RS – Maringá, 16ª RS – Apucarana, 17ª RS – Londrina, 18ª RS – Cornélio Procópio, 19ª RS – Jacarezinho, 20ª RS – Toledo, 21ª RS – Telêmaco Borba e 22ª RS – Ivaiporã.^(6)^

A 2ª RS abrange 29 municípios, incluindo Curitiba.^(6)^ Entretanto, os dados dos serviços administrados pela prefeitura foram obtidos e analisados de forma independente. Isso ocorre porque Curitiba é um município de gestão plena que responde diretamente à Coordenação Estadual de Infecções Sexuais Transmissíveis/AIDS e, com frequência, diretamente ao Ministério da Saúde, tendo seus dados disponibilizados separadamente no sistema.^(6)^ Dessa forma, os resultados da 2ª RS contemplam as informações dos demais 28 municípios.

Na análise estatística, foi considerada a hipótese de autocorrelação de primeira ordem entre as proporções observadas, devido ao fato de se tratar praticamente de uma mesma população acompanhada ao longo do tempo. Para essa análise, foi utilizado o teste de Durbin-Watson e as proporções foram corrigidas pela estatística de Prais-Winsten.

Com o intuito de estimar o acréscimo ou o decréscimo do índice de supressão viral ao longo do período analisado, foi calculada a variação percentual estimada, a partir do ajuste da regressão linear ao logaritmo natural das proporções. Foram consideradas significativas as variações na proporção desses índices quando valor de p≤0,05. O pacote estatístico utilizado foi o Stata versão 14.

O estudo obteve aprovação do Comitê de Ética em Pesquisa da Universidade Federal do Paraná (UFPR), de acordo com o parecer 2.620.673/2018, CAAE: 82936318.3.0000.0102, e do Comitê de Ética em Pesquisa do Hospital do Trabalhador/Secretaria do Estado da Saúde do Paraná, de acordo com o parecer 2.674.606/2018; CAAE: 82936318.3.3001.5225. Por ser desenvolvido a partir de dados secundários, não foi necessária a assinatura do Termo de Consentimento Livre e Esclarecido (TCLE).

## RESULTADOS

Entre as pessoas vivendo com HIV/AIDS no Paraná no período do estudo predominavam homens (60,0%), entre 30 e 49 anos (49,5%), brancos (61,6%) e com 4 a 11 (51,2%) anos de estudo (Tabela 1). A razão obtida entre os sexos masculino e feminino foi de 1,5.

**Tabela 1:** Características sociodemográficas da população em terapia antirretroviral no estado do Paraná

Variáveis		n (%)
Sexo		
	Masculino	19.241 (60,0)
	Feminino	12.822 (40,0)
	Não informado	33 (0,1)
Faixa etária, anos		
	<5	1.069 (3,3)
	5-19	650 (2,0)
	20-29	5.083 (15,8)
	30-39	7.651 (23,8)
	40-49	8.250 (25,7)
	50-59	6.221 (19,4)
	≥60	3.172 (9,9)
Cor da pele		
	Branca	19.783 (61,6)
	Parda	5.112 (15,9)
	Preta	1.403 (4,7)
	Amarela	140 (0,4)
	Indígena	31 (0,1)
	Não informada	5.627 (17,5)
Escolaridade, anos		
	Nenhum	1.569 (4,9)
	1-3	2.211 (6,9)
	4-7	8.147 (25,4)
	8-11	8.285 (25,8)
	ou mais	3.985 (12,4)
	Não informada	7.899 (24,6)
Total		32.096 (100,0)

Em janeiro de 2019, no final do período abordado por este estudo, 34.472 pessoas tinham diagnóstico de HIV no estado do Paraná, das quais 93,12% encontravam-se em tratamento antirretroviral. Encontravam-se no *gap* de tratamento 2.376, pois não tinham iniciado o uso dos antirretrovirais.

Em janeiro de 2018, início do período de observação, 91,4% das pessoas em tratamento antirretroviral já apresentavam carga viral ≤1.000 cópias/mL no estado do Paraná (93,3±2,0%). Entretanto, existem diferenças importantes nos valores de carga viral entre as RS do estado (Tabela 2).

**Tabela 2:** Percentagem de pacientes com carga viral ≤1.000 cópias/mL, coeficientes de regressão e variações no estado do Paraná e Regionais de Saúde, entre janeiro de 2018 e janeiro de 2019

Regional de Saúde (RS)	2018 (%)						2019 (%)	Coeficiente	IC95%	Valor de p	Variação (%)	IC95%	Interpretação
	Janeiro	Março	Maio	Julho	Setembro	Novembro	Janeiro						
1ª Paranaguá	87,7	87,8	88,3	92,0	92,3	93,5	94,4	1,38	0,9-1,8	0,001	3,55	2,86-4,24	Aumento
2ª Metropolitana de Curitiba	92,2	91,8	92,5	94,9	95,1	96,1	96,9	1,00	0,7-1,3	<0,001	2,48	2,06-2,90	Aumento
3ª Ponta Grossa	89,0	89,0	88,8	91,2	92,0	93,2	95,6	1,46	0,9-2,0	0,002	3,66	2,77-4,55	Aumento
4ª Irati	94,5	93,3	91,4	94,9	94,5	95,0	95,9	0,61	-0,0-1,2	0,055	1,51	0,54-2,49	Estabilidade
5ª Guarapuava	87,5	88,2	88,5	92,1	93,5	94,2	94,7	1,46	0,9-2,0	0,002	3,74	2,78-4,72	Aumento
6ª União da Vitória	87,0	86,5	87,0	92,6	92,1	94,7	95,6	1,89	1,3-2,5	0,001	4,91	3,94-5,89	Aumento
7ª Pato Branco	86,3	86,1	85,4	90,4	91,3	93,8	94,8	1,95	1,2-2,7	0,002	5,11	3,94-6,30	Aumento
8ª Francisco Beltrão	91,7	91,4	92,2	93,5	93,9	95,7	97,2	1,23	0,8-1,6	0,001	2,98	2,40-3,56	Aumento
9ª Foz do Iguaçu	92,1	91,6	92,8	94,4	94,9	96,3	97,1	1,05	0,9-1,2	<0,001	2,59	2,41-2,77	Aumento
10ª Cascavel	91,9	91,4	92,0	93,8	93,6	93,5	96,9	0,72	0,4-1,0	0,004	1,80	1,29-2,31	Aumento
11ª Campo Mourão	88,2	87,9	88,1	91,8	92,5	93,9	94,0	1,38	0,8-1,9	0,002	3,55	2,71-4,40	Aumento
12ª Umuarama	90,4	89,9	91,0	94,7	94,7	95,2	95,7	1,18	0,5-1,8	0,008	2,97	1,91-4,04	Aumento
13ª Cianorte	76,8	77,0	80,2	90,2	87,6	88,2	91,9	2,75	0,7-4,7	0,019	7,81	4,1-11,6	Aumento
14ª Paranavaí	92,5	92,6	92,9	95,0	93,6	94,6	97,5	0,67	0,2-1,1	0,011	1,65	1,0-2,3	Aumento
15ª Maringá	91,1	91,2	92,6	94,0	93,3	94,0	96,5	0,79	0,4-1,2	0,004	1,98	1,4-2,6	Aumento
16ª Apucarana	85,5	84,7	84,9	90,1	89,9	91,6	95,2	1,95	1,3-2,6	0,001	5,17	4,1-6,2	Aumento
17ª Londrina	89,8	89,4	89,9	92,0	93,0	93,5	95,8	1,23	0,9-1,5	<0,001	3,11	2,6-3,6	Aumento
18ª Cornélio Procópio	84,8	84,9	83,7	87,1	85,9	89,9	93,2	1,86	0,4-3,3	0,023	4,83	2,6-7,1	Aumento
19ª Jacarezinho	83,2	82,7	82,4	89,5	89,0	90,2	92,9	2,02	1,2-2,9	0,003	5,51	4,0-7,0	Aumento
20ª Toledo	86,5	85,6	86,5	88,8	87,6	91,3	94,0	1,61	0,7-2,5	0,007	4,16	2,8-5,5	Aumento
21ª Telêmaco Borba	85,4	85,5	86,6	89,0	88,6	88,7	89,5	0,72	0,1-1,3	0,028	1,91	0,9-2,9	Aumento
22ª Ivaiporã	84,8	83,6	84,2	88,5	88,9	91,7	93,6	2,05	1,5-2,6	<0,001	5,46	4,6-6,3	Aumento
Prefeitura Municipal de Curitiba	94,9	94,5	94,9	95,8	96,1	96,7	97,7	0,65	0,5-0,8	<0,001	1,56	1,3-1,8	Aumento
Paraná	91,4	91,1	91,6	93,8	94,0	94,9	96,4	1,01	0,8-1,2	<0,001	2,52	2,1-2,9	Aumento

Teste de Durbin-Watson e proporções corrigidas pela estatística de Prais-Winsten. Foram significativos valores de p≤0,05.

IC95%: intervalo de confiança de 95%.

A maior média de pacientes com carga viral inferior a 1.000 cópias/mL foi obtida pela Região de Saúde da Prefeitura Municipal de Curitiba (95,8±1,1%), enquanto a menor média esteve na 13ª RS (84,5±6,4%). A tabela 2 apresenta a proporção bimestral de pacientes em terapia antirretroviral com carga viral ≤1.000 cópias/mL em todas as RS do Paraná. A figura 1 ilustra a evolução das RS e do estado neste indicador.

**Figura 1:** Evolução da percentagem de pacientes com carga viral ≤1.000 cópias/mL no estado do Paraná e em suas Regionais de Saúde, entre os meses de janeiro de 2018 a janeiro de 2019

PMC: Prefeitura Municipal de Curitiba.

Os coeficientes de regressão revelaram tendência de aumento do percentual de pessoas com supressão virológica em praticamente todas as RS (p<0,05), exceto na 4ª RS, cuja tendência é de estabilidade (p=0,055), para a qual a percentagem de pacientes com carga viral ≤1.000 cópias/mL superou a média do estado.

Na análise de carga viral ≤50 cópias/mL, o Paraná alcançou o índice de 90% das pessoas em terapia antirretroviral com supressão viral em novembro de 2018, próximo ao final do período de observação (janeiro de 2019). Entretanto, considerando todo o período do estudo, a média se manteve abaixo de 90% (88,6±2,9%). A maior média de pacientes com carga viral inferior a 50 cópias/mL foi obtida na região de saúde da Prefeitura Municipal de Curitiba (92,5±1,6%), enquanto a menor média esteve na 13ª RS (78,3±6,6%) (Tabela 3). A figura 2 ilustra a evolução das RS e do estado nesse indicador.

**Tabela 3:** Percentagem de pacientes com carga viral ≤50 cópias/mL, coeficientes de regressão e variações no estado do Paraná e Regionais de Saúde, entre janeiro de 2018 e janeiro de 2019

Regional de Saúde (RS)	2018 (%)						2019 (%)	Coeficiente	IC95%	Valor de p	Variação (%)	IC95%	Interpretação
	Janeiro	Março	Maio	Julho	Setembro	Novembro	Janeiro						
1ª Paranaguá	82,6	82,0	81,8	85,8	86,4	88,0	89,4	1,56	1,1-2,1	0,001	4,30	3,4-5,2	Aumento
2ª Metropolitana de Curitiba	86,0	85,7	86,7	90,3	90,7	92,2	93,2	1,53	1,1-1,9	<0,001	4,02	3,3-4,7	Aumento
3ª Ponta Grossa	82,2	81,7	81,3	85,5	86,7	88,3	91,5	2,08	1,4- 2,8	0,001	5,68	4,5-6,9	Aumento
4ª Irati	89,0	86,7	85,0	87,7	87,6	87,1	89,8	0,67	-0,1-1,5	0,081	1,77	0,5-3,1	Estabilidade
5ª Guarapuava	78,8	78,8	79,9	83,3	86,0	87,7	88,1	2,07	1,4-2,7	0,001	5,88	4,7-7,0	Aumento
6ª União da Vitória	80,7	79,7	80,3	84,3	82,5	87,3	92,9	2,74	0,9-4,6	0,014	7,27	4,4-10,2	Aumento
7ª Pato Branco	77,9	78,6	78,2	83,3	84,4	89,0	89,9	2,54	1,8-3,3	0,001	7,21	5,9-8,6	Aumento
8ª Francisco Beltrão	84,9	83,2	84,0	86,8	87,4	89,1	91,6	1,69	1,3-2,1	<0,001	4,52	3,9-5,2	Aumento
9ª Foz do Iguaçu	85,6	85,4	87,1	89,0	89,4	90,8	92,5	1,29	1,1-1,5	<0,001	3,41	3,1-3,7	Aumento
10ª Cascavel	87,7	86,9	87,9	90,2	89,5	89,7	93,3	0,88	0,4-1,3	0,005	2,30	1,6-3,0	Aumento
11ª Campo Mourão	82,2	80,6	81,9	86,5	86,4	88,5	90,6	1,91	1,4-2,4	<0,001	5,28	4,4-6,2	Aumento
12ª Umuarama	84,9	84,0	85,4	90,6	90,5	91,4	92,4	1,70	0,9-2,5	0,005	4,55	3,1-6,0	Aumento
13ª Cianorte	71,3	70,8	73,1	82,1	80,3	82,9	87,8	3,08	2,0-4,2	0,001	9,53	7,3-11,8	Aumento
14ª Paranavaí	88,4	87,4	88,6	91,1	90,6	92,0	95,2	1,29	0,8-1,8	0,002	3,32	2,6-4,1	Aumento
15ª Maringá	86,3	86,3	87,8	91,0	90,4	91,3	94,3	1,34	0,9-1,8	0,001	3,51	2,8-4,3	Aumento
16ª Apucarana	76,7	75,6	76,3	83,1	84,4	86,9	90,2	2,98	2,2-3,8	<0,001	8,68	7,1-10,2	Aumento
17ª Londrina	83,2	82,6	83,3	87,2	88,1	89,0	91,6	1,74	1,3-2,2	<0,001	4,73	3,9-5,5	Aumento
18ª Cornélio Procópio	76,3	76,1	76,7	82,3	81,0	85,0	86,4	2,04	1,5-2,5	<0,001	5,98	5,0-6,9	Aumento
19ª Jacarezinho	76,0	74,9	75,7	83,8	84,0	85,5	87,6	2,61	1,5-3,7	0,003	7,72	5,6-9,9	Aumento
20ª Toledo	80,7	79,7	81,0	84,9	83,7	86,8	91,2	1,95	1,2-2,7	0,002	5,39	4,1-6,7	Aumento
21ª Telêmaco Borba	78,3	78,6	79,1	79,1	80,3	80,9	83,0	1,86	0,3-3,4	0,030	5,25	2,5-8,0	Aumento
22ª Ivaiporã	75,0	73,9	73,7	81,3	84,1	87,5	90,4	3,65	2,5-4,8	0,001	10,80	8,6-13,1	Aumento
Prefeitura Municipal de Curitiba	91,0	90,5	91,3	92,7	93,1	93,8	95,0	0,84	0,7-1,0	<0,001	2,12	1,9-2,3	Aumento
Paraná	85,8	85,3	86,2	89,3	89,7	90,9	92,8	1,44	1,1-1,8	<0,001	3,79	3,3-4,3	Aumento

Teste de Durbin-Watson e proporções corrigidas pela estatística de Prais-Winsten. Foram significativos os valores de p≤0,05.

IC95%: intervalo de confiança de 95%.

**Figura 2:** Evolução da percentagem de pacientes com carga viral ≤50 cópias/mL no estado do Paraná e em suas Regionais de Saúde, entre os meses de janeiro de 2018 a janeiro de 2019

PMC: Prefeitura Municipal de Curitiba.

A proporção de pacientes com carga viral ≤50 cópias/mL aumentou significativamente no estado do Paraná (p<0,001), em Curitiba (p<0,001) e em 21ª RS. Excetuando-se a 4ª RS do estado (p=0,081), cujos valores (87,6±1,6%) se aproximam da média do estado (88,6±2,9%). Houve variação de 3,79% (3,25%-4,34%) no Paraná, indicando menor homogeneidade dos resultados em relação ao índice anterior (≤1.000 cópias/mL). O coeficiente de regressão de 1,44 indica uma tendência de aumento desse índice no estado (p<0,001).

## DISCUSSÃO

Com o propósito de verificar o atendimento à meta 90-90-90 da UNAIDS/OMS,^(3)^ este estudo investigou o quantitativo de pessoas diagnosticadas com HIV no estado do Paraná, a proporção das pessoas em tratamento antirretroviral, e, entre estas, a proporção de pessoas com carga viral suprimida.

Entre as 32.096 pessoas em tratamento antirretroviral no estado do Paraná no período do estudo, predominou o sexo masculino (59,95%), em conformidade com outros estudos.^(7,8)^ A razão entre os sexos masculino e feminino na população em tratamento foi de 15 homens para cada 10 mulheres (masculino:feminino = 1,5). Ao se comparar com dados estaduais e nacionais da população diagnosticada com o HIV, observa-se que as razões correspondem a 21 homens para cada dez mulheres (masculino:feminino = 2,1) no estado do Paraná e a 26 homens para cada 10 mulheres (masculino:feminino = 2,6) no Brasil.^(9)^ Portanto, a proporção entre os diagnosticados não é mantida entre os usuários da terapia antirretroviral, com maior prejuízo para o sexo masculino. Considerando a recomendação de tratar todas as pessoas diagnosticadas, essa diferença pode sugerir dificuldades no acesso ou na adesão ao tratamento antirretroviral pelas pessoas do sexo masculino.^(9)^Segundo a UNAIDS, os homens são menos propensos a fazer o teste para o HIV e a buscar o tratamento antirretroviral. As causas para esse comportamento ainda não estão bem esclarecidas, mas parecem envolver conceitos empíricos relacionados à masculinidade.^(10)^

A faixa etária predominante da população em terapia antirretroviral no Paraná foi entre 30 e 49 anos (49,54%), conforme identificado em estudo anterior.^(11)^ Entretanto, no Brasil e no Paraná, os novos casos de infecção pelo HIV ocorrem predominantemente entre pessoas de 20 a 39 anos.^(9,12)^ Essa faixa etária, de modo geral, é mais suscetível a comportamentos de risco e à infecção pelo HIV, e isso pode representar breve uma modificação da faixa etária das pessoas em tratamento para o HIV.^(12)^

Observou-se, ainda, uma maior prevalência de brancos (61,64%), seguidos de pardos (15,93%). Esse resultado foi confirmado por outros estudos e está em consonância com as características demográficas da população do Paraná.^(12-14)^

Em relação à escolaridade, o elevado percentual de casos ignorados (24,6%) dificultou uma melhor avaliação, porém mais da metade da população em tratamento antirretroviral no Paraná tem entre 4 e 11 anos de escolaridade, semelhantemente a outros estudos do Paraná e do Brasil.^(9,12)^

O Brasil tem sido reconhecido mundialmente por sua forte política de combate à epidemia do HIV/AIDS. Como resultado, o país já havia diagnosticado 85% de todas as pessoas vivendo com HIV em seu território no ano de 2017,^(15)^ ou seja, um quantitativo semelhante ao obtido no estado do Paraná em 2015.^(16)^

Por meio das informações obtidas no Sistema de Controle de Exames Laboratoriais (SISCEL) e no SICLOM, obteve-se que a proporção de pessoas diagnosticadas com HIV em tratamento antirretroviral correspondeu a 93,12% dos diagnosticados no Paraná. Portanto, no período do estudo, o estado já cumpria satisfatoriamente com a segunda meta da UNAIDS, referente ao fornecimento da terapia a 90% da população com HIV.^(3)^ Vale destacar que o Ministério da Saúde propõe o início precoce da terapia antirretroviral, logo após o diagnóstico da infecção pelo HIV, independente do estágio clínico e imunológico das pessoas infectadas, pois considera os benefícios na redução da morbimortalidade, a diminuição da transmissão da infecção e o impacto na redução da tuberculose, além da disponibilidade de opções terapêuticas mais cômodas e bem toleradas.^(5)^ Dessa forma, a prática do início precoce da terapia antirretroviral proporciona um *gap* de tratamento reduzido em todo o país.

Diversos estudos fortalecem a necessidade da oferta do tratamento antirretroviral à população infectada. Estudo realizado na África do Sul demonstrou que o aumento de 1% na cobertura do tratamento antirretroviral gerou redução de 1,1% na incidência do HIV.^(17)^ De forma complementar, estudo canadense demonstrou redução de 1,2% na incidência do HIV a cada 1% de aumento no número de pessoas com supressão viral decorrente da terapia.^(18)^

Considerando o ponto de corte de 1.000 cópias do RNA viral/mL de sangue, proposto pela UNAIDS/OMS,^(3)^ verificou-se que o Paraná alcançou a meta de 90% de pessoas em tratamento com supressão viral desde o início do período analisado (janeiro de 2018). Esse índice foi mantido ao longo de um ano do estudo, e a tendência de aumento foi confirmada estatisticamente.

No ponto de corte mais crítico, proposto pelo PCDT do Ministério da Saúde,^(5)^ o percentual de pacientes com carga viral ≤50 cópias do RNA viral/mL de sangue foi alcançado mais recentemente no estado (novembro de 2018), entretanto demonstra tendência de aumento no Paraná. Esses números sugerem um bom resultado das ações de aceleração do combate ao HIV, bem como o sucesso da terapia antirretroviral na população no Paraná. Esses êxitos provavelmente decorrem da recomendação de se tratarem todas as pessoas vivendo com HIV, independente do status imunológico, da disponibilização de sistemas que facilitam o monitoramento dos pacientes (como o SIMC) e do incentivo da Secretaria de Estado da Saúde do Paraná para o uso desses sistemas e para prescrição de esquemas mais potentes desde a primeira linha de tratamento antirretroviral, que incluem novos medicamentos, como o dolutegravir.^(5,19,20)^

Em 2015, dados epidemiológicos do Ministério da Saúde já demonstravam que o Brasil atingira o parâmetro de 90% de supressão viral proposto pela meta 90-90-90. No ano de 2016, o país apresentava 91% das pessoas em terapia antirretroviral com resultados de carga viral menores que 1.000 cópias/mL. Em 2017, esse índice subiu para 92%, sendo esse resultado semelhante ao encontrado no atual estudo realizado no Paraná.^(9)^

No entanto, os estudos que indicam a não transmissão do HIV por pacientes com supressão viral adotaram como ponto de corte uma carga viral menor que 200 cópias/mL.^(2)^ Ademais, estudos recentes revelaram que grande proporção de pacientes com carga viral inferior a 1.000 cópias/mL, porém superiores a 80 cópias/mL, abrigam mutações de resistência a medicamentos antirretrovirais, as quais conduzem ao fracasso da terapia e à transmissão do vírus resistente.^(21)^

Dessa forma, o Ministério da Saúde do Brasil considera pacientes em sucesso virológico aqueles com carga viral inferior a 50 cópias/mL, o que indica que a replicação viral está suprimida, sem transmissão do vírus ou progressão da doença.^(22)^

No Paraná, os serviços da Prefeitura Municipal de Curitiba, das Regionais de Paranavaí (14ª RS) e de Maringá (15ª RS) e da região Metropolitana de Curitiba (2ª RS) apresentaram os melhores resultados de sucesso virológico do estado no período avaliado. No entanto, ainda existem RS distantes do índice almejado, como as de Cianorte (13ª RS), Telêmaco Borba (21ª RS) e Cornélio Procópio (18ª RS).

Esses resultados podem ser explicados, ao menos em parte, pelas diferenças na estrutura dos serviços de saúde e nas questões socioeconômicas (como escolaridade e renda) de cada Regional. Tais fatores geram diferentes níveis de vulnerabilidade que impactam no acesso e na adesão à terapia antirretroviral e, consequentemente, no sucesso virológico do tratamento. Essa relação foi identificada por diversos estudos.^(21-26)^

Apesar das diferenças dentro do estado, os coeficientes de regressão revelaram tendência de aumento do percentual de pessoas com carga viral indetectável em quase todas as RS, exceto na 4ª RS, cuja tendência é de estabilidade. Outros estudos obtiveram resultados semelhantes^(27,28)^ ou inferiores de supressão viral.^(29,30)^ Isso demonstra que, de forma geral, a política de saúde e os serviços disponibilizados no Paraná têm tido êxito no que tange ao sucesso virológico nas pessoas em terapia antirretroviral.

Importante ressaltar que, após alcançar a supressão viral em 90% dos pacientes em terapia antirretroviral, é necessário manter a carga viral suprimida, para reduzir a transmissão do vírus HIV, traduzindo-se, assim, em benefícios para a saúde coletiva.^(20,22)^

Ressalta-se ainda que este estudo considerou em sua avaliação o segundo e terceiro estágios da meta 90-90-90, que são, respectivamente, porcentagem de pacientes em tratamento e pacientes com supressão viral. Entretanto, a cascata de cuidado do HIV contempla intervenções dependentes umas das outras, de forma que se não houver avanços no parâmetro do diagnóstico, o primeiro estágio da meta, os demais pontos da cascata não representarão a realidade. Por esse motivo, é importante monitorar todos os pontos da cascata do cuidado contínuo do HIV. Neste contexto, estima-se que 80% das pessoas vivendo com HIV no Brasil sabem que são infectadas, mas não existem informações específicas sobre o Paraná.^(15)^

## CONCLUSÃO

O estado do Paraná alcançou o segundo e terceiro parâmetros da meta 90-90-90 do Programa Conjunto das Nações Unidas sobre HIV/AIDS (UNAIDS) da Organização Mundial da Saúde. Isso demonstra que a política de saúde voltada à população vivendo com HIV/AIDS e os serviços de saúde disponibilizados no Paraná, apesar das diferenças regionais, têm obtido êxito em parâmetros relevantes para o controle da epidemia. Entretanto, os parâmetros são interdependentes, e tornam-se fundamentais alcançar e manter o primeiro parâmetro da meta, que consiste em diagnosticar 90% das pessoas infectadas pelo HIV na população. Será necessária, assim, uma melhor estruturação da Rede de Atenção, a qual exigirá investimentos em educação em saúde e em estratégias para aumentar a demanda por serviços de testagem e aconselhamento em HIV.
